# Tanshinone analog inhibits castration-resistant prostate cancer cell growth by inhibiting glycolysis in an AR-dependent manner

**DOI:** 10.1016/j.jbc.2024.107139

**Published:** 2024-03-05

**Authors:** Jia Yu, Shengyou Li, Sha Cheng, Mashaal Ahmad, Chao Chen, Xinwei Wan, Shinan Wei, Weidong Pan, Heng Luo

**Affiliations:** 1 State Key Laboratory of Functions and Applications of Medicinal Plants, Guizhou Medical University, Guiyang, PR China; 2 Institute of Pharmacology and Bioactivity, Natural Products Research Center of Guizhou Province, Guiyang, PR China; 3 School of Pharmaceutical Sciences, Guizhou University, Guiyang, PR China; 4 Department of Pharmacy, Guizhou Hospital of the First Affiliated Hospital, Sun Yat-sen University, Guiyang, PR China

**Keywords:** Castration-resistant prostate cancer, glycolysis, AR/PKM2 axis, danshinone IIA derivative, pyruvic acid

## Abstract

Androgen receptor (AR) is one of the key targets for the treatment of castration-resistant prostate cancer (CRPC). Current endocrine therapy can greatly improve patients with CRPC. However, with the change of pathogenic mechanism, acquired resistance often leads to the failure of treatment. Studies have shown that tanshinone IIA (TS-IIA) and its derivatives have significant antitumor activity, and have certain AR-targeting effects, but the mechanism is unknown. In this study, the TS-IIA analog TB3 was found to significantly inhibit the growth of CRPC *in vitro* and *in vivo*. Molecular docking, cellular thermal shift assay, and cycloheximide experiments confirmed that AR was the target of TB3 and promoted the degradation of AR. Furthermore, TB3 can significantly inhibit glycolysis metabolism by targeting the AR/PKM2 axis. The addition of pyruvic acid could significantly alleviate the inhibitory effect of TB3 on CRPC cells. Besides, the knockdown of AR or PKM2 also could reverse the effect of TB3 on CRPC cells. Taken together, our study suggests that TS-IIA derivative TB3 inhibits glycolysis to prevent the CRPC process by targeting the AR/PKM2 axis.

Prostate cancer (PCa) is a common malignancy within the male reproductive system, currently regarded as a significant medical concern for men. It is the most common solid tumor in both Europe and the United States, with the case numbers surpassing those of lung or colorectal cancer, and thus the foremost tumor jeopardizing men's health (1). Although PCa incidence is lower in China than in European and American countries, the increasing average life expectancy and the Westernization of the diet have led to noticeable increases in cases with PCa and related mortality (2). Inhibition of the androgen receptor (AR) signaling through androgen deprivation therapy is the mainstay treatment for PCa. Nevertheless, approximately 40% to 60% of patients with PCa experience relapse after first-line treatment and develop castration-resistant prostate cancer (CRPC), which is a relatively more aggressive and lethal form of the disease (3).

AR is a ligand-dependent transcription factor in the nuclear receptor family and is one of the major receptors for androgens. After binding to ligands such as dihydrotestosterone (DHT), AR forms a homodimer, which transfers to the nucleus, recognizes the androgen response elements of downstream gene promoters, and promotes the transcription and expression of downstream genes (4). Studies have shown that abnormal expression of AR or aberrant activity of associated signaling pathways is the main pathological mechanism underlying CRPC. Accordingly, strategies targeting the AR pathway, use of AR antagonists, and suppression of AR expression, have been employed to treat CRPC (5). However, despite the partial extension of survival observed in many patients with CRPC treated with drugs targeting the AR pathway, drug resistance often ensues, ultimately leading to treatment failure (6, 7). Therefore, it is necessary to study the mechanisms underlying CRPC and find novel AR-targeting therapeutics.

In a population-based retrospective study involving 40,692 men diagnosed with PCa, patients treated with *Salvia miltiorrhiza* showed a 5 to 10% higher survival rate than patients who were untreated, and this protective effect was positively correlated with both the dose and duration of *S. miltiorrhiza* administration (8). *S. miltiorrhiza* is a widely used Chinese medicinal herb in China, and its main bioactive ingredients are the lipid-soluble tanshinone compounds, particularly tanshinone IIA (TS-IIA), dihydrotanshinone, tanshinone I, and cryptotanshinone (9). Numerous studies have shown that danshinone regulates various molecular pathways in PCa, including signal transducer and activator of transcription 3 pathway, AR pathway, PI3K/AKT/mTOR pathway, mitogen-activated protein kinase pathway, thus affecting the release of proinflammatory cytokines, cell proliferation, apoptosis, and tumor metabolism (10, 11, 12, 13). Besides, it has been reported that TS-IIA analogs can be used as AR inhibitors (14). TS-IIA derivative PTS33 showed the effects of AR inhibition, blocking AR-regulated gene expression, and cell growth inhibition in AR-positive PCa cells (15). Thus, TS-IIA compounds have high potential in clinical applications against PCa by targeting AR.

In our previous studies, we fused TS-IIA and *β*-lapachone to obtain the parent nucleus of the tanshinone analog by a strategy of fusion design. On this basis, we have derived a series of chemical compounds. Among them, TB3 was obtained by introducing the aromatic ring fragment at the benzyl position of the furan ring (16). It was found that the novel tanshinone analog, TB3, significantly inhibits the proliferation and cell cycle of PCa cells, and has lower cytotoxicity to human normal liver than other tanshinone analogs. However, the mechanisms underlying these effects are unclear. Thus, this study aimed to explore the mechanisms whereby the tanshinone analog TB3 inhibits CRPC cell survival, ultimately providing a rationale for the application of tanshinone analogs to treat CRPC.It was found that the new tanshinone analog TB3 significantly inhibited PCa cell proliferation, upregulated the oxidative stress process, and had lower cytotoxicity than other tanshinone analogs, but the mechanism of this TB3 against PCa is not clear. This study aimed to explore the mechanism of tanshinone analog TB3 in inhibiting CRPC cell survival and provide a rationale for targeting tanshinone analogs of AR to treat CRPC.

## Results

### Danshinone analog TB3 inhibits CRPC cell survival

**Figure 1:**
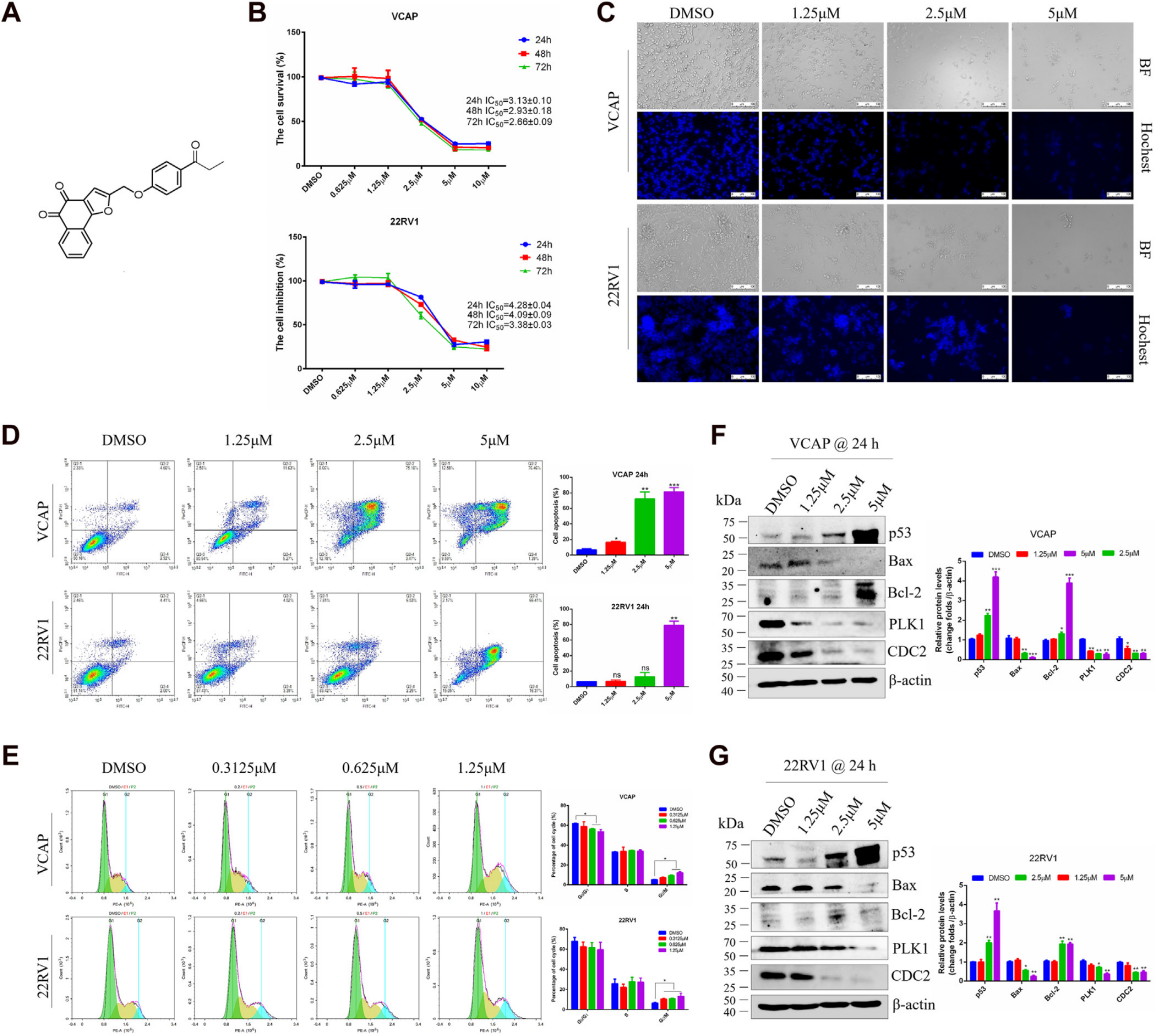
**TB3 inhibits CRPC cell survival.***A*, chemical structure of TB3 (2-(((4-propionyl)oxy)methyl)naphtho[1,2-b]furan-4,5-dione); *B*, cell inhibition of VCAP and 22RV1 cells under TB3 treatment by MTT assay. *C*, morphology and Hoechst staining of VCAP and 22RV1 cells. The scale bar represents 100 *μ*m; Apoptosis (*D*) and cell cycle (*E*) of VCAP and 22RV1 cells upon TB3 treatment; the levels of apoptosis- and cell cycle-related proteins in TB3-treated VCAP (*F*) and 22RV1 (*G*) cells. ∗*p* < 0.05, ∗∗*p* < 0.01. All the data represent the mean ± SD from three independent experiments. VCAP, vertebral cancer of the prostate.

The chemical structure of TB3 is shown in Figure 1*A*. Pharmacological analysis of TB3 by employing a concentration gradient showed that when the final concentration of TB3 was 5 *μ*M or 10 *μ*M, cell proliferation viability was significantly reduced. The cell survival of vertebral cancer of the prostate (VCAP) and 22RV1 reached 50% and 20% in the 2.5-*μ*M TB3 treatment for 24 h (Fig. 1*B*). For the following experiment, the concentrations (0 μM，1.25 μM，2.5 μM，and 5 μM) and time course (24 h) were chosen according to the IC_50_ dose and time course. Morphological features under a light microscope, Hoechst staining, cell apoptosis, cell cycle, and western-blotting results revealed that TB3 significantly promoted cell apoptosis and blocked cell cycle in the G_2_/M phase, and those effects were concentration-dependent (Fig. 1, *C*–*G*). In summary, the results confirm that the tanshinone analog TB3 exhibits notable *in vitro* anti-CRPC effects.

### TB3 regulates glycolysis metabolism in CRPC

**Figure 2:**
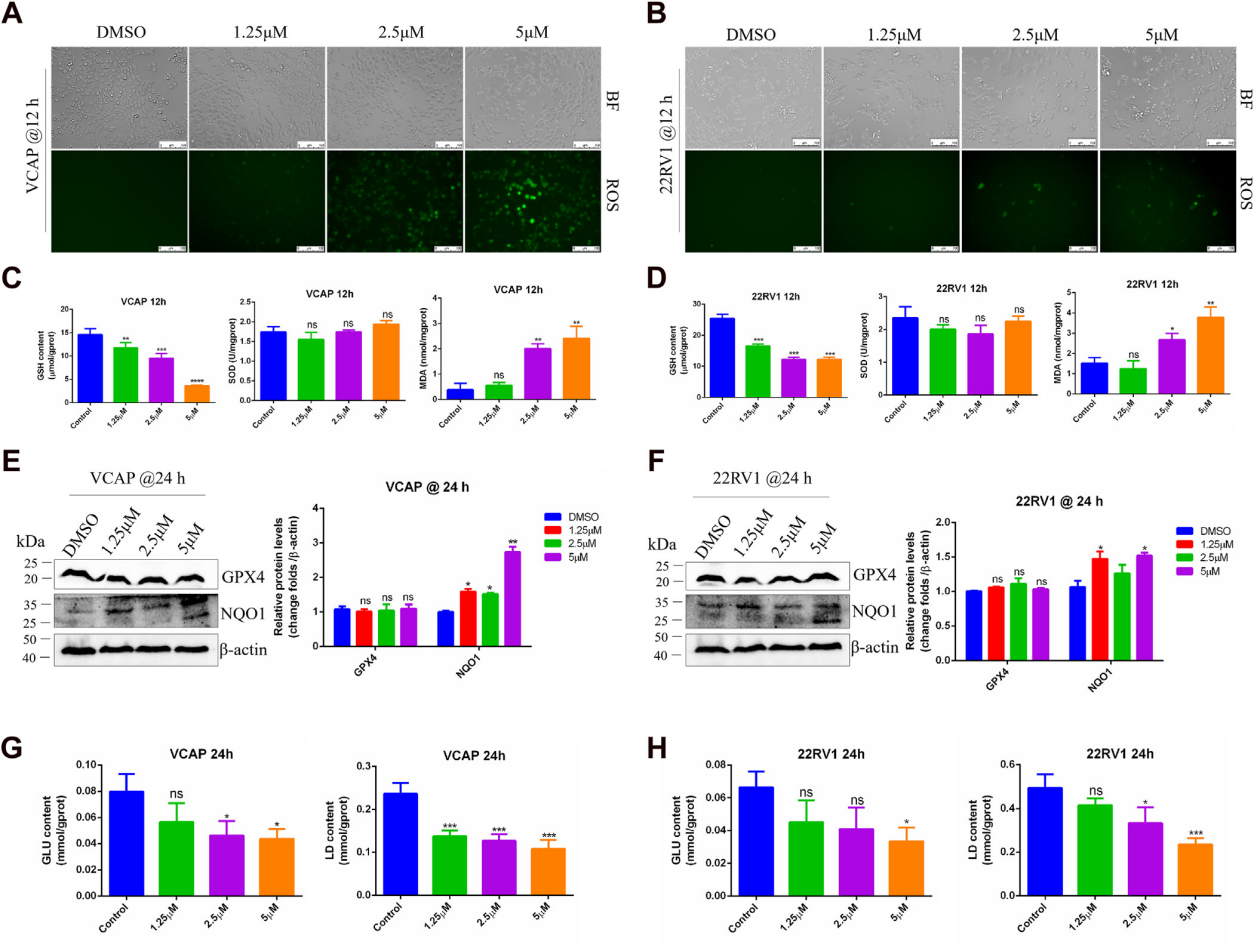
**TB3 may regulate glycolysis metabolism in CRPC cells.***A* and *B*, the bright-field images and ROS levels of TB3-treated VCAP and 22RV1 cells. The scale bar represents 100 *μ*m. *C* and *D*, the GSH, SOD, and MDA levels of VCAP and 22RV1 cells treated with TB3 for 12 h. *E* and *F*, GPX4 and NQO1 protein levels were detected using western blotting after 24 h of TB3 treatment. The GLU and LD levels in VCAP (*G*) and 22RV1 (*H*) cells treated with TB3 for 24 h. ∗*p* < 0.05, ∗∗*p* < 0.01, ∗∗∗*p* < 0.001. All the data represent the mean ± SD from three independent experiments. GLU, glucose; LD, lactate; MDA, malondialdehyde. VCAP, vertebral cancer of the prostate.

To explore whether the mechanism of TB3 inhibits cell survival in CRPC cells, reactive oxygen species (ROS) (17) and ferroptosis were detected. CRPC cells were treated with TB3 for 12 h, and the results related to ROS levels indicated a significant increase in oxidative stress preceding apoptosis, with VCAP cells exhibiting higher TB3 sensitivity than 22RV1 cells at the same concentration (Fig. 2, *A* and *B*). This observation suggests that TB3 induces oxidative damage in CRPC cells. Additionally, an assessment of other indicators of cellular oxidative damage revealed that after 12 h of TB3 treatment, glutathione and malondialdehyde were significantly upregulated and downregulated, respectively, with no notable change in superoxide dismutase levels (Fig. 2, *C* and *D*). Besides, western-blot results revealed upregulation in NQO1 levels at 24 h, whereas GPX4 levels remained unaffected, indicating that TB3-induced cell death is likely unrelated to ferroptosis (Fig. 2, *E* and *F*). These findings collectively suggest that TB3 increases oxidative stress in CRPC cells but does not induce ferroptosis.

Except for ferroptosis, oxidative stress has been reported to affect many processes such as glucose metabolism and cellular microenvironment (18). To elucidate the molecular mechanism whereby TB3 promotes CRPC cell death, we investigated the glucose metabolism of CRPC cells following treatment with TB3 for 24 h. The results showed a significant decrease in the glucose level and the lactate level at 24 h (Fig. 2, *G* and *H*). These findings indicate that TB3 reprograms the glucose metabolism in CRPC cells.

### TB3 inhibits the key enzyme expression of glycolysis metabolism

**Figure 3:**
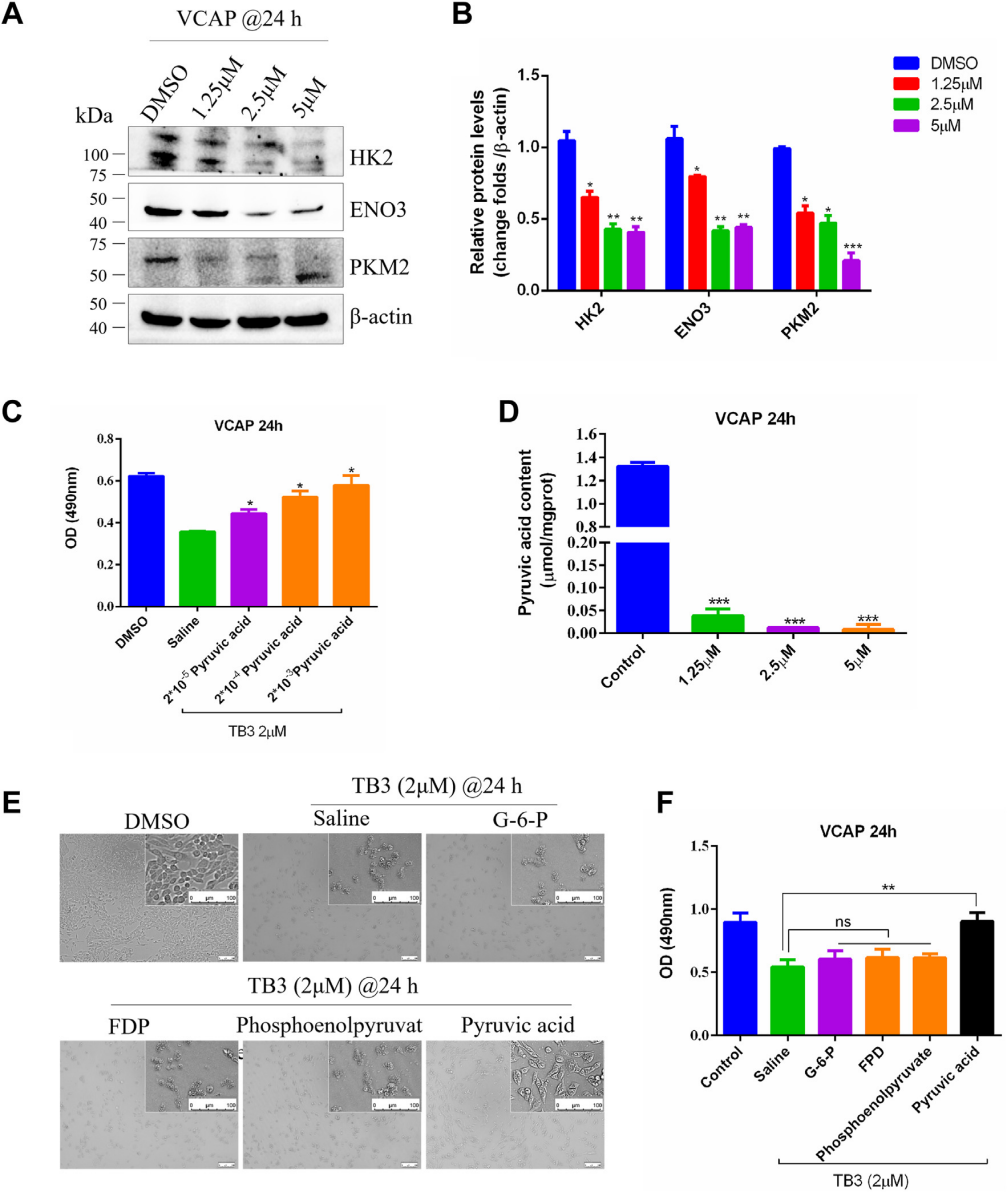
**TB3 suppresses glycolysis metabolism in CRPC cells.***A* and *B*, cellular levels of various key proteins involved in glycolysis were assessed using western blotting after 24 h of TB3 treatment. *C*, viability of the cells treated with 2 *μ*M TB3 and different concentrations of pyruvic acid, as assessed using the MTT assay. *D*, pyruvic acid level in VCAP treated by TB3 for 24 h. *E* and *F*, rescue of TB3-treated VCAP cells by intermediates of the glycolysis pathway. The scale bar represents 100 *μ*m. ∗*p* < 0.05, ∗∗*p* < 0.01, ∗∗∗*p* < 0.001. All the data represent the mean ± SD from three independent experiments. VCAP, vertebral cancer of the prostate.

To further explore the specific mechanism in accordance with which TB3 regulates the glucose metabolism in CRPC cells, the expression levels of key rate-limiting enzymes in the glycolytic pathway, namely, HK2, PFK, and PKM2, were assessed using western blotting. The results showed that HK2, PFK, and PKM2 were significantly downregulated by TB3 (Fig. 3, *A* and *B*).

To further confirm the key glycolysis kinases by TB3 regulation, downstream products of HK2, PFK, ENO3, and PKM2, such as glucose-6-phosphate (G-6-P), fructose-1,6-diphosphate, phosphoenol-pyruvic acid, and pyruvic acid, were introduced to TB3**-**treated cells to assess whether any of these products could rescue the cytostatic effect of TB3. The rescue results of different concentrations revealed that only pyruvic acid could do so (other data do not show) (Fig. 3*C*). Furthermore, quantification of cellular pyruvic-acid content using a kit revealed significant downregulation of pyruvic acid in TB3**-**treated CRPC cells (Fig. 3*D*), confirming that TB3 primarily reduces pyruvic-acid production by downregulating PKM2, thereby suppressing the proliferation of CRPC cells.

Additionally, the introduction of pyruvic acid effectively rescued the TB3-induced inhibition of cell proliferation, but not other intermediates of the glycolysis pathway (Fig. 3, *E* and *F*). These findings collectively suggest that TB3 primarily suppresses glycolysis by downregulating the key rate-limiting enzyme PKM2, leading to reduced pyruvic-acid production, and consequently suppressing the proliferation of CRPC cells.

### AR is a potential target of TB3 against CRPC cells

To further explore the molecular mechanism whereby TB3 suppresses glycolysis in CRPC cells, we predicted the targets of TB3. Initially, SwissTargetPrediction revealed 107 possible binding targets. Subsequently, 1666 CRPC-related targets and 4684 glycolysis-related targets from GeneCards, OMIM, and Disgenet databases were identified. By performing a Venn analysis, 28 common targets were identified (Fig. S1*A*). The 28 targets were then subjected to protein–protein interaction networks analysis (Fig. S1*B*). Among the top 10 targets in the protein–protein interaction results, AR exhibited the highest binding energy with TB3 (Fig. S1*C*). Molecular-docking results illustrated that TB3 can form hydrogen bonds with multiple residues (VAL A: 685, VAL A: 684, and TRP A: 651) of human AR (2q7i), contributing to structural stability (Fig. S1*D*).

**Figure 4:**
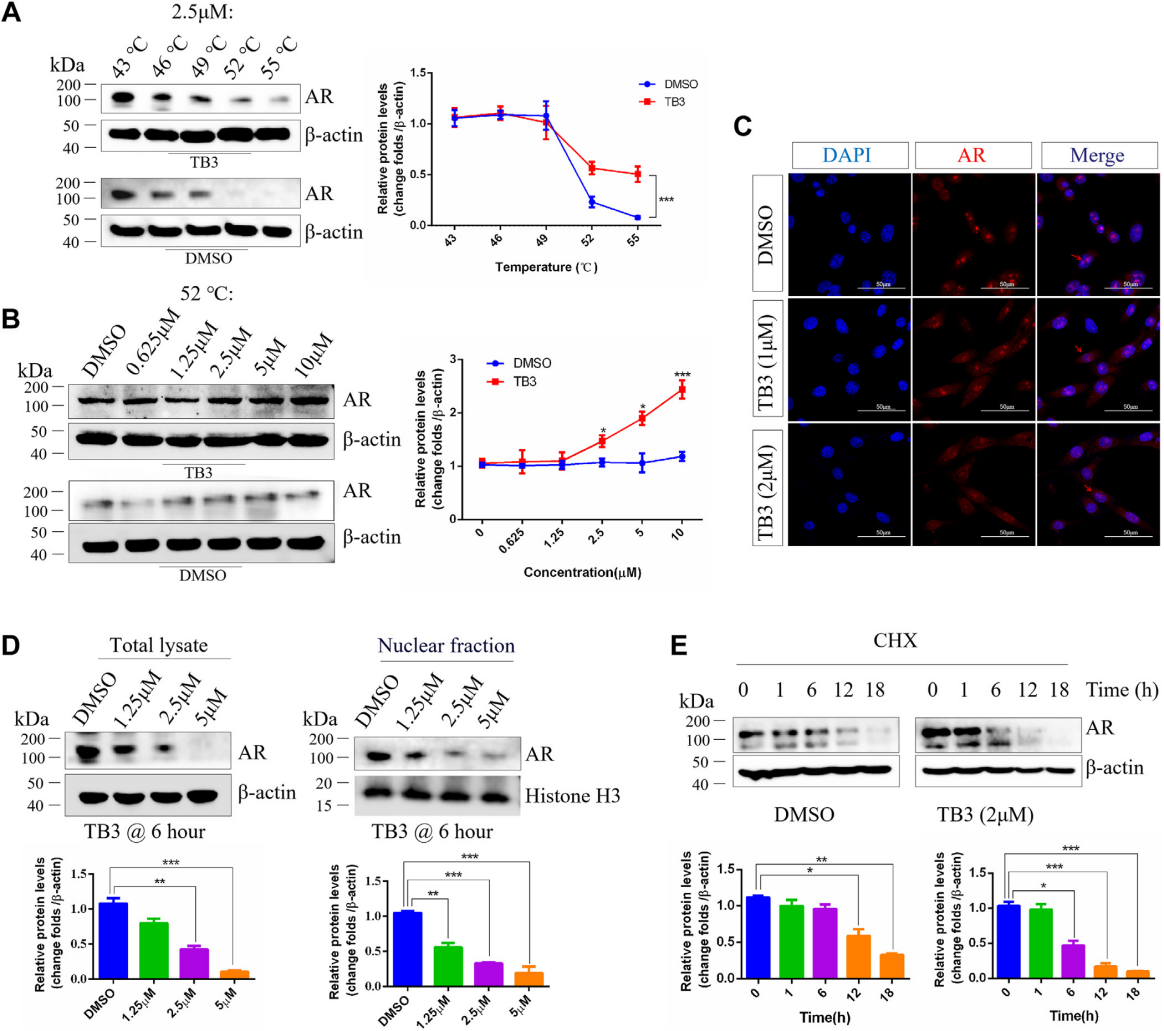
**TB3 as a novel AR antagonist in CRPC cells.** Cellular thermal shift assay of TB3 with a temperature gradient (*A*) and concentration gradient (*B*) in VCAP cells, as assessed *via* western blotting. *C*, subcellular localization pattern of AR in TB3-treated VCAP cells, as assessed *via* immunofluorescence. The scale bar represents 50 *μ*m. *D*, western blot analysis of the AR levels of total lysate and nuclear fraction in VCAP cells treated with TB3 at different concentration for 6 h. *E*, western-blotting analysis of AR level in VCAP cells with/without 3 μM TB3 were treated with 50 μg/ml CHX for 0, 1, 6, 12, or 18 h. ∗*p* < 0.05, ∗∗*p* < 0.01, ∗∗∗*p* < 0.001. All the data represent the mean ± SD from three independent experiments. VCAP, vertebral cancer of the prostate.

Cellular thermal shift assay (CETSA) was discovered to study drug-target binding (19). The TB3 binding to AR was detected by CETSA, and the results confirmed that TB3 binding to AR was more stable at 52 °C compared to control groups and had a dependent concentration gradient (Fig. 4, *A* and *B*). Besides, the immunofluorescence results showed that 1 *μ*M TB3 inhibited the nucleoplasmic translocation of AR (Fig. 4*C*), while the nucleoplasmic translocation and expression of AR were also affected by TB3 in 2 *μ*M treatment for 24 h (Fig. 4*C*). Moreover, TB3 significantly inhibited AR expression in a concentration-gradient-dependent manner between total lysate and nuclear fraction (Fig. 4*D*). In addition, the cycloheximide test was used to detect the stability of AR protein after TB3 treatment, and the results showed that 2 μM TB3 reduced the half-life of AR in VCAP cells (Fig. 4*E*). In summary, these findings suggest that AR is a potential target of TB3 against CRPC cell.

### TB3 regulates the CRPC cell survival by targeting the AR/PKM2 axis

**Figure 5:**
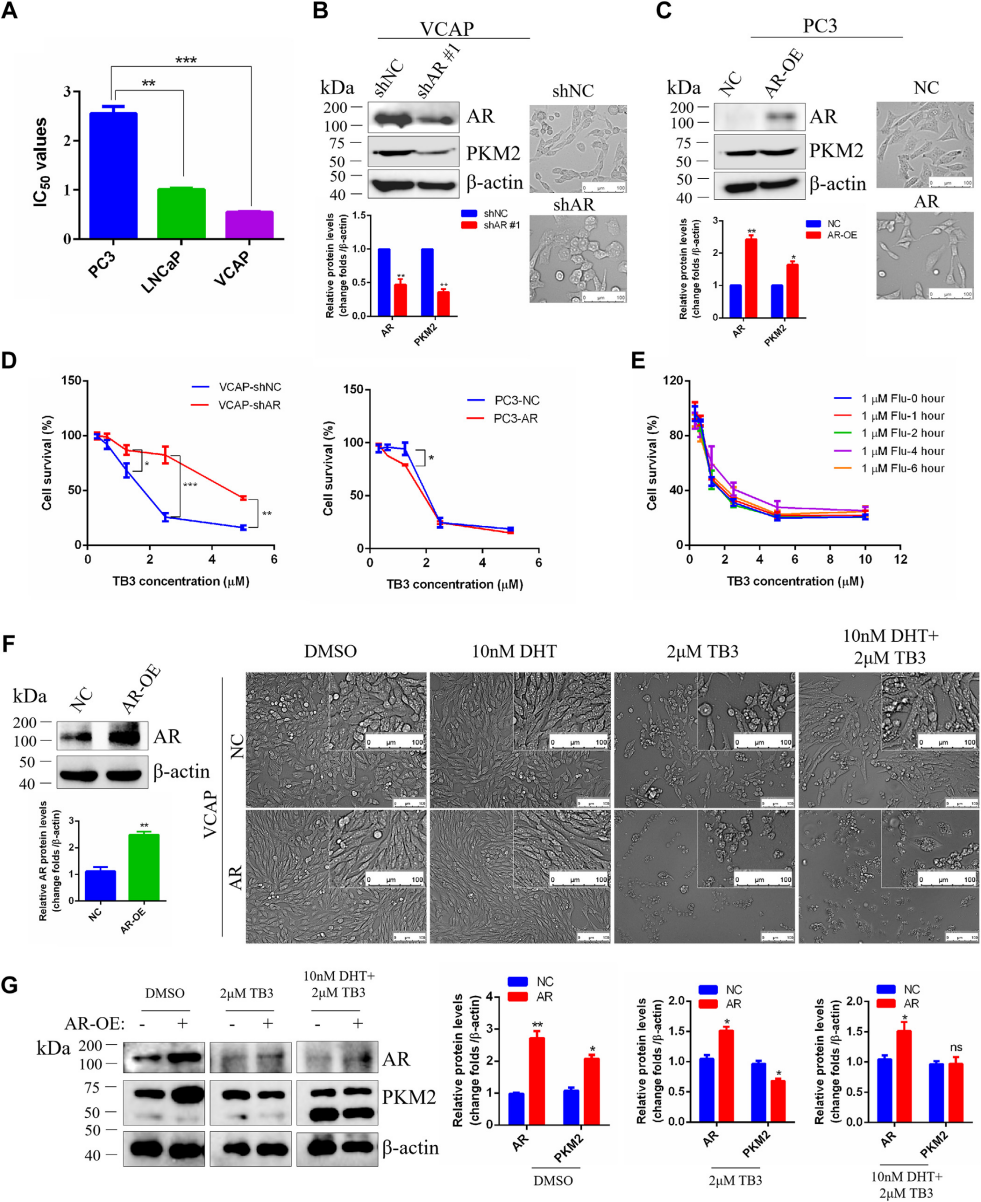
**TB3 downregulates PKM2 by targeting AR.***A*, IC_50_ values in different prostate cancer cell lines with varying degrees of AR expression. Western-blot analysis and bright-field images after AR inhibition (shAR#1) in VCAP cells (*B*) and AR overexpression (AR-OE) in PC3 cells (*C*). The scale bar represents 100 *μ*m. *D*, MTT-assay results demonstrating the TB3 sensitivity of AR inhibition VCAP cells and AR-OE PC3 cells. *E*, MTT assay of TB3 on VCAP cells after flutamide (FLU) pretreated cells. *F*, the western-blot analysis of AR-OE VCAP cells and the bright-field images of 2 *μ*M TB3- or 10 nM DHT-treated cells transfected AR for 24 h in VCAP. The scale bar represents 100 *μ*m. *G*, western-blot analysis of AR and PKM2 proteins in 2 *μ*M TB3- or 10 nM DHT-treated cells transfected AR for 24 h. ∗*p* < 0.05, ∗∗*p* < 0.01, ∗∗∗*p* < 0.001. All the data represent the mean ± SD from three independent experiments. VCAP, vertebral cancer of the prostate.

The TB3 sensitivity of PCa cell lines with varying levels of AR expression was investigated. It was found that VCAP and LNCaP cells, which express AR, exhibited greater TB3 sensitivity than PC3 cells, which lack AR expression (Fig. 5*A*). To elucidate the molecular mechanism of TB3 suppression glycolysis, AR was inhibited in VCAP (Fig. 5*B*) and was overexpressed in PC3 cells (Fig. 5*C*). MTT assay revealed TB3 was more sensitive in AR overexpression PC3 cells than that in the control group. Consistent with this, TB3 was less sensitive in the downregulation of AR (shAR #1) VCAP cells than in the control group (Fig. 5*D*). Furthermore, the inhibition of TB3 on VCAP cells decreased after the AR antagonist flutamide-pretreated cells for 4 h, but there was no significant difference (Fig. 5*E*).

**Figure 6:**
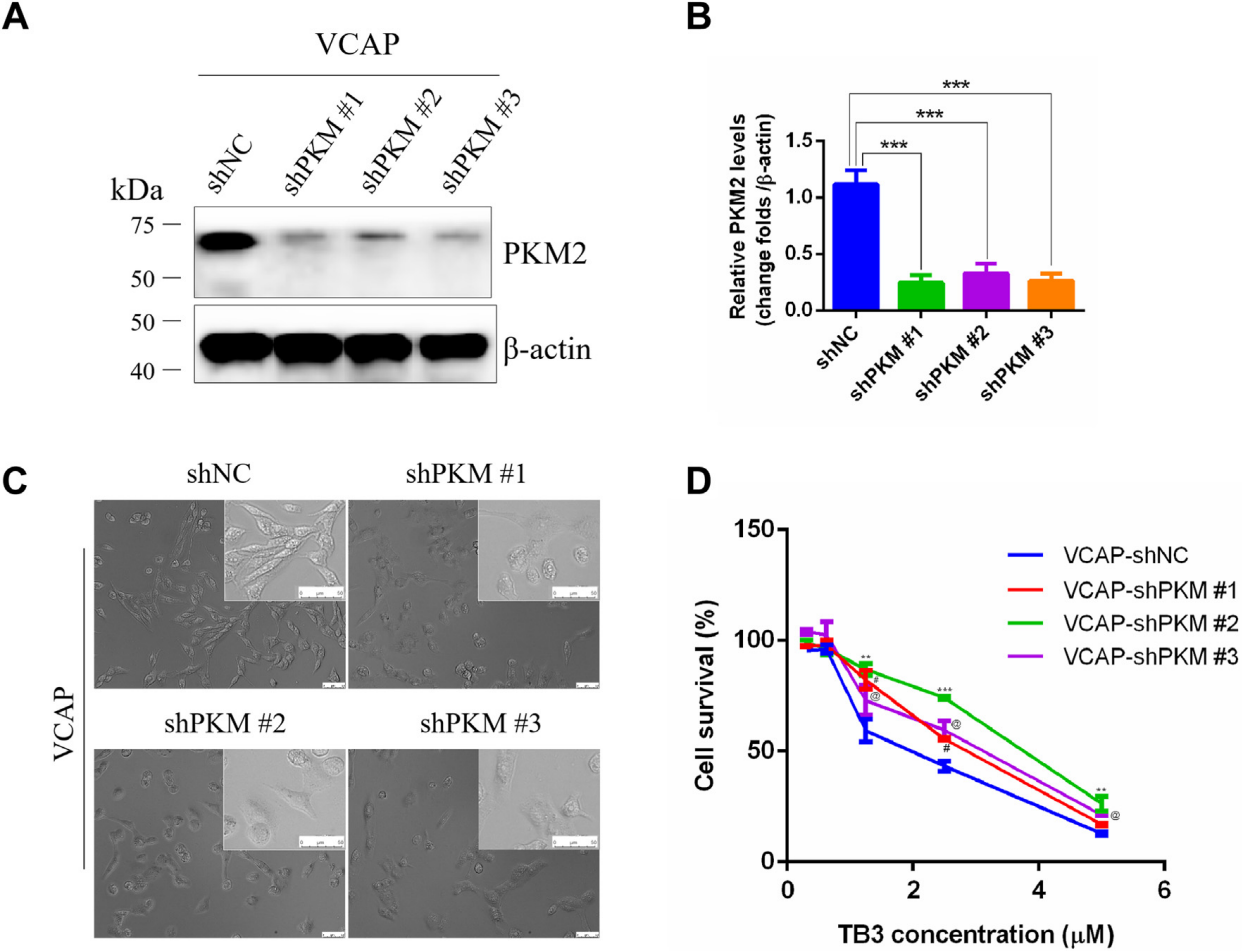
**TB3 downregulates PKM2 by targeting AR.***A* and *B*, western-blot analysis and light-field images (*C*) after PKM2 inhibition (shPKM#1, shPKM#2, and shPKM#3) in VCAP cells. The scale bar represents 50 *μ*m. *D*, MTT assay results demonstrating the TB3 sensitivity of PKM2 inhibition VCAP cells. ∗*p* < 0.05, ∗∗*p* < 0.01, ∗∗∗*p* < 0.001. All the data represent the mean ± SD from three independent experiments. VCAP, vertebral cancer of the prostate.

As a ligand of AR, DHT plays a pivotal role in AR function. It was observed that DHT significantly changed the morphology of VCAP cells and alleviated the cell death caused by TB3 compared with no DHT (Fig. 5*D*). Moreover, VCAP cells overexpressing AR were more sensitive to TB3 than NC controls, where the expression of PKM2 was significantly suppressed (Fig. 5, *D* and *E*). In contrast, the simultaneous addition of 10 nM DHT and 2 *μ*M TB3 to cells transfected with the AR significantly reversed the death of VCAP cells, with an increasing PKM2 expression in the process (Fig. 5, *D* and *E*). To further validate PKM2 as the downstream target gene of TB3, the knockdown of PKM2 was performed in VCAP cells and the downregulation of PKM2 could indeed reverse the inhibitory effect of TB3 on VCAP cells (Fig. 6). The above results suggest that DHT acts in concert with AR to promote AR-mediated transcriptional activation of the key genes with AREs an upregulate PKM2 protein expression. However, TB3 reduced AR nuclear localization and degradation, downregulated PKM2 expression, and then inhibited the glycolysis and CRPC processes. These findings collectively suggest that TB3 regulates glycolysis and cell survival by targeting the AR/PKM2 axis.

### TB3 inhibits CRPC growth through AR/PKM2 signal *in vivo*

**Figure 7:**
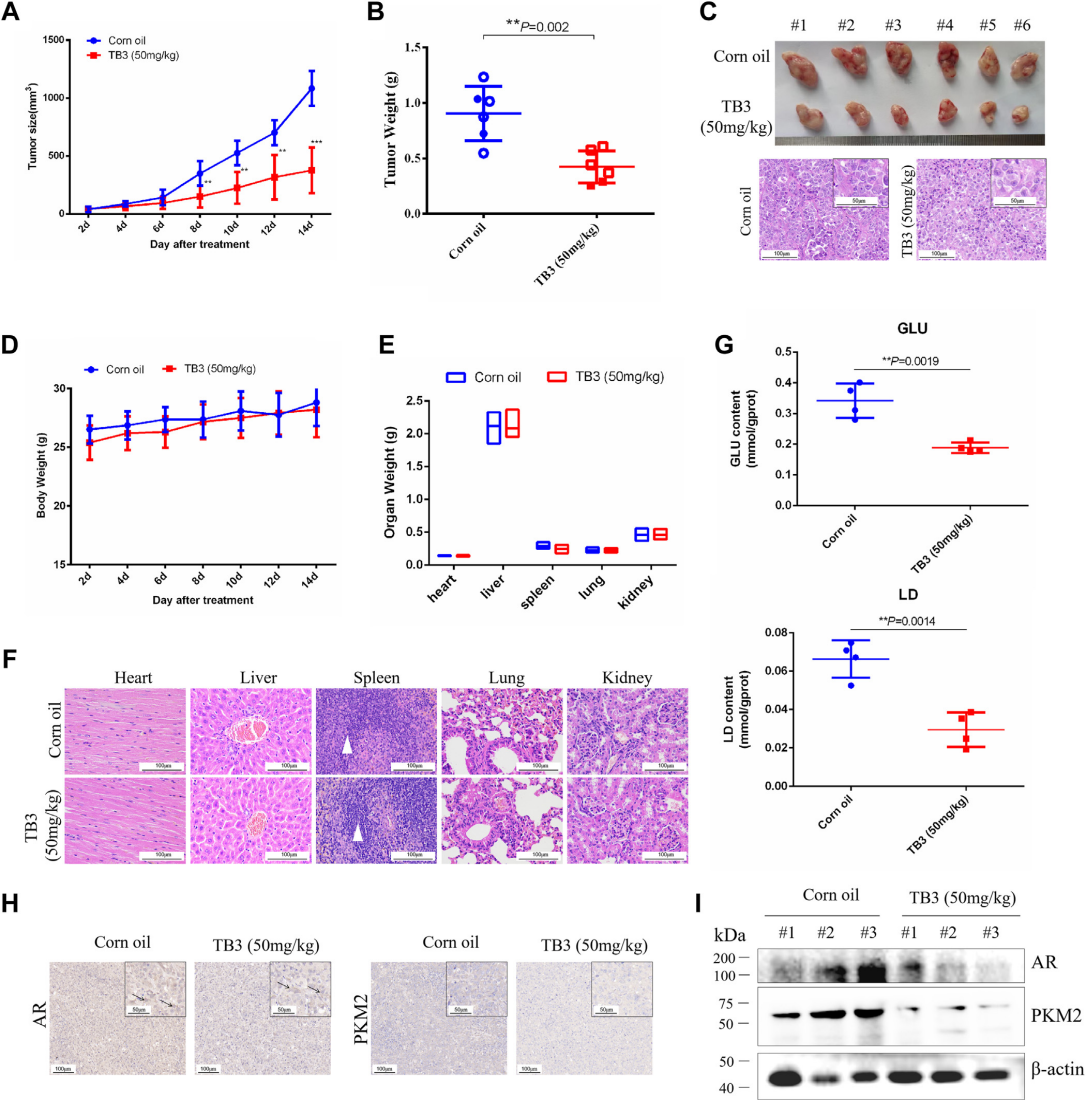
**TB3 suppresses the growth of xenotransplanted CRPC cells by inhibiting glycolysis in an AR-dependent manner.***A*, volume changes after 2 weeks of 50 mg/kg TB3 treatment. *B*, tumor quality. *C*, tumor tissue and hematoxylin–eosin (H&E) staining. *D*, body weight. *E*, weight of visceral tissues. *F*, H&E staining of visceral tissues. *White triangle*: white pulp of the spleen. *G*, GLU and LD content in tumor tissues. *H*, immunohistochemical and (*I*) western-blot analyses of the tumor in each group for AR and PKM2 expression. The scale bar represents 100 *μ*m. ∗*p* < 0.05, ∗∗*p* < 0.01. All the data represent the mean ± SD from three independent experiments. GLU, glucose; LD, lactate.

**Figure 8:**
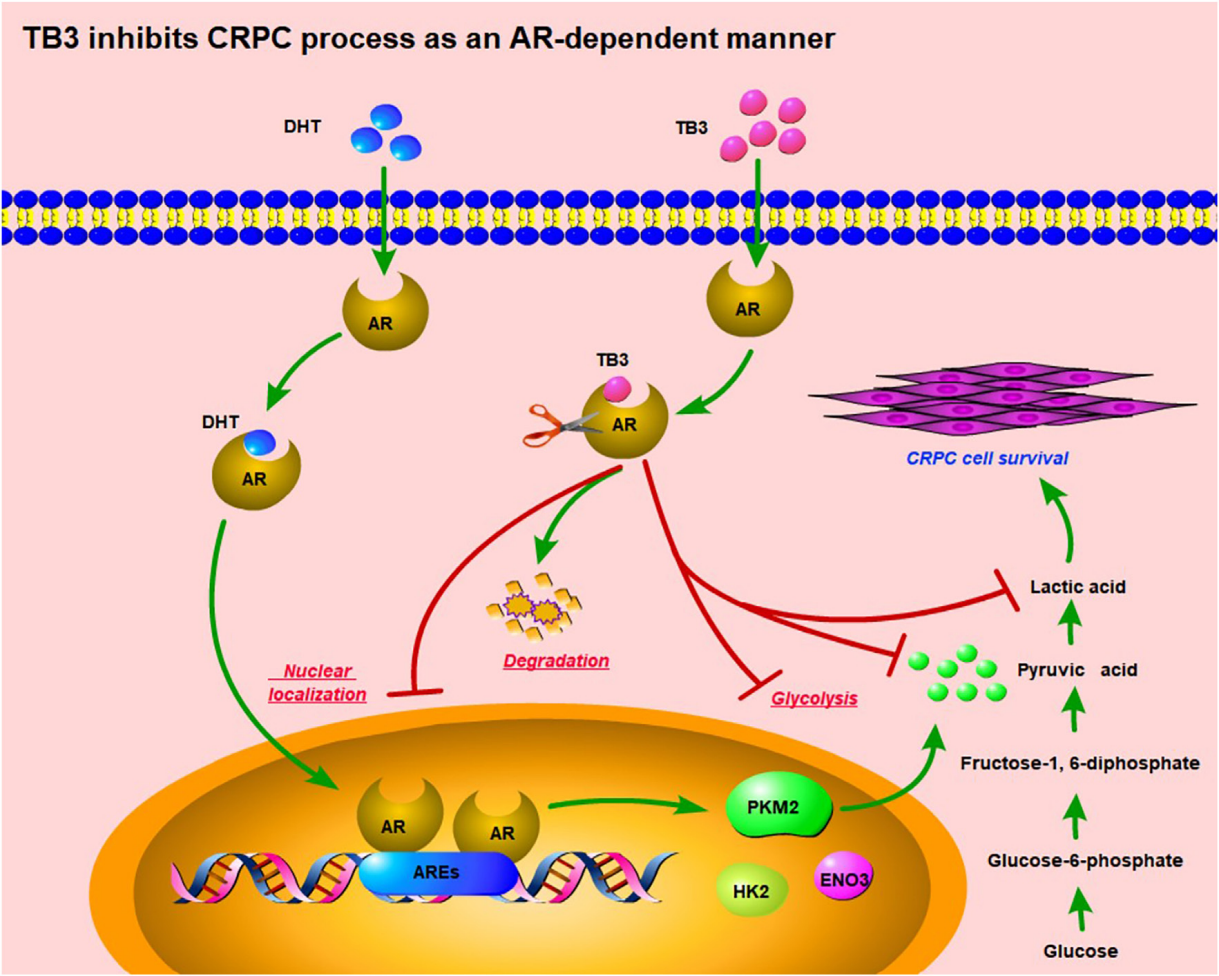
**TB3 inhibitsCRPC progress by targeting AR/PKM2 axis.**

Subcutaneous xenotransplantation of CRPC cells to nude mice substantiated that TB3 (50 mg/kg) exerted a significant inhibitory effect on CRPC growth *in vivo*, evidenced by a significant reduction in tumor volume and weight compared with the model group (Fig. 7, *A* and *B*). Notably, TB3 treatment resulted in a marked decrease in the number of hyperplastic cells within the tumor (Fig. 7*C*). Importantly, no significant alterations were observed in terms of body weight or the weight of visceral tissues (Fig. 7, *D* and *E*). Hematoxylin-eosin staining revealed no TB3-induced visceral tissue damage in heart, liver, lung, and kidney tissues, while the lymphocytes in the spleen white pulp of TB3-treated were significantly increased, compared with the model group (Fig. 7*F*). Furthermore, immunohistochemistry and western-blot analyses demonstrated that TB3 significantly downregulated AR and PKM2 levels, concurrently inhibiting nucleoplasmic translocation of AR (Fig. 7, *G* and *H*). The abovementioned results indicate that TB3 inhibits CRPC progress by inhibiting glycolysis in an AR-dependent manner (Fig. 8).

## Discussion

In the treatment of CRPC, primarily one of the therapeutic strategies is still to target the AR pathway. Nevertheless, it is noteworthy that a significant proportion of patients with CRPC ultimately develop drug resistance following treatment targeting this pathway, leading to treatment failure (5, 6). Tanshinone compounds are a class of broad-spectrum bioactive compounds with antitumor effects in most human cancer cells, such as leukemia (20), colorectal cancer (21), and breast cancer (22). However, in PCa, tanshinones seem to have a particular advantage over other cancers (8). In our study, TB3 was found to significantly inhibit the growth of CRPC *in vitro* and *in vivo* (Figs. 1 and 7). Besides, the antitumor activity of TB3 against CRPC may also be related to the immune response due to an increase in the white pulp of the spleen (Fig. 7*F*), which may be the reason why *S. miltiorrhiza* has a certain protective effect on patients with PCa (8), but The effect of TB3 on the immune response was not thoroughly explored in this study.

It is reported that TS-IIA inhibits PCa cell growth by inhibiting AR expression (23). In our study, TB3 inhibited AR nuclear localization and promoted AR degradation in a concentration gradient-dependent manner (Fig. 4), which is consistent with previous studies (24). Besides, AR expression was positively correlated with TB3 sensitivity in PCa cells with different AR expressions (Fig. 5), indicating that the tanshinone analog TB3 is a novel AR antagonist. Additional studies have shown that as a potent antagonist of AR, 4,4-dimethyl on the A ring of TS-IIA is important for the antiandrogenic and maspin induction of TS-IIA (23). In contrast, our target compound TB3 does not have 4,4-dimethyl on the A ring of TS-IIA, but it can still play a similar role. Therefore, we believe that the parent nuclear structure of TS-IIA is the core structure of targeted inhibition of AR, but this difference was not analyzed in depth in this study.

Glycolysis represents a prevalent metabolic pathway in cancer cells and is a prominent target in cancer therapy. Although there is limited research on the effects of tanshinones on glycolysis in CRPC, the modulation of glycolysis by TS-IIA has been extensively investigated in other cancers. Tan-IIA plays a regulatory role in suppressing glycolysis by downregulating PI3K/AKT/mTOR and JNK signaling pathways (25), decreasing HK2 (26), inhibiting the activities of intracellular AKT/mTOR and HIF-1α pathways (27). In our study, we cotreated CRPC cells with TB3 and four key metabolic intermediates, namely, G-6-P, fructose-1,6-diphosphate, phosphoenolpyruvic acid, or pyruvic acid. Remarkably, only pyruvic acid could rescue the TB3-induced cell death (Figs. 2 and 3). Interference with AR can directly affect PKM2 expression (Fig. 5), whereas knockdown of PKM2 reversed the inhibitory effect of TB3 on VCAP cells (Fig. 6). In addition, the cellular morphology of AR inhibition was consistent with that of PKM2 inhibition, with VCAP cells changing from a smaller spindle to a larger polygon (Figs. 5*B* and 6*C*). Our study demonstrates that the AR/PKM2 axis is a very important target for CRPC treatment, and the TB3-regulated glycolysis by AR/PKM2 axis, thereby inhibiting the malignant progression of CRPC.

## Experimental procedures

### Cell culture

Human PCa PC3, VCAP, 22RV1, LNCaP, and 293T cell lines were obtained from a typical culture conservation center (American Type Culture Collection) and stored in a special liquid-nitrogen freezing tank at the Key Laboratory of Natural Products Chemistry, Chinese Academy of Sciences. Cells were cultured in Dulbecco's modified Eagle's medium supplemented with 10% fetal bovine serum (BI) and 1% penicillin and streptomycin (Beyotime), and incubated at 37 °C with 5% CO_2_, 95% air, and 95% humidity.

### Lentivirus packaging and transfection

The plasmids were constructed by Hunan Fenghui Biotechnology Co, Ltd. All target plasmids, such as pcDNA3.1-AR (NM_000044), shAR #1, shPKM #1, shPKM #2, and shPKM #3 (NM_002654), were mixed with packaging plasmids (psPAX2) and envelope plasmids (pMD2G) at the ratio of 2:2:1, respectively, and added into 293T cells for virus packaging. Collection and filtration of lentivirus was performed at 24 h, 48 h, and 72 h. Cells were placed in 6-well plates, and the culture medium (containing 10% fetal bovine serum) was changed 2 to 3 h before transfection. Cell morphology and fluorescence were observed using an inverted microscope after transfection for 24 h (Leica). Stably transfected cells were identified *via* puromycin selection. The shAR #1 sequence: 5′-GGAGCTCTCACATGTGGAAGC-3′; shPKM #1 sequence: 5′-GCTGTGGCTCTAGACACTAAA-3′; shPKM #2 sequence:5′-GTTCGGAGGTTTGATGAAATC-3′; shPKM #3 sequence:5′-CGGGTGAACTTTGCCATGAAT-3′.

### Animal experiment

All animal experiments are conducted according to the principles of the International Guidelines for the Care and Use of Laboratory Animals. Male BALB/c-null mice (Spelford Laboratory Animal Co, Ltd) were used for animal experiments. VCAP cells in a good growth state were collected, divided into 1 × 10^7^ cells/ml with normal saline, and placed on ice. Each mouse was injected with 100-*μ*l cell suspension subcutaneously. One week after inoculation, the tumor length and width of mice were observed and recorded. When the tumor volume of nude mice was about 100 mm^3^, they were randomly divided into a model group and a treatment group (50 mg/kg). TB3 was given intragastrically every other day for 2 weeks. The weight of the mice and the length (a) and width (b) of the tumors were measured every 2 days. Tumor volume = 0.5 × a × b^2^). After 14 days, the mice were dissected, and the heart, liver, spleen, lung, kidney, and tumor were taken and weighed.

### MTT assay

22RV1 and VCAP CRPC cells were cultured as described above and collected when they reached the logarithmic growth phase. They were counted, and then 5000 to 10,000 cells/well were seeded into 96-well plates. The drug concentration gradient included 0.625 *μ*M, 1.25 *μ*M, 2.5 *μ*M, 5 *μ*M, and 10 *μ*M. All the cells were incubated for 24 h, 48 h, and 72 h at 37 °C in a CO_2_ incubator. Then, they were incubated with the MTT dye solution (Beyotime) for 4 h. Afterward, the culture supernatant was removed, and the cells were incubated with 150 *μ*l dimethyl sulfoxide (DMSO) (Beyotime)/well in a 37 °C shaker for 15 min. Finally, the absorbance at 490 nm was measured using a microplate reader (Gene), and the rate of inhibition of cell proliferation was calculated using the following formula:

[1-(experimental group absorbance–blank group absorbance)/(control group absorbance–blank group absorbance)] × 100%

### Annexin V-FITC propyl iodide assay

Cell suspensions were prepared as described above and then seeded into 6-well plates at a density of 100,000 to 200,000 cells/well. After treatment with various concentrations of TB3 (1.25 *μ*M, 2.5 *μ*M, and 5 *μ*M; the vehicle DMSO alone was used for 0 *μ*M TB3) for 48 h, the cells were collected, washed with pre-cooled phosphate-buffered saline (PBS) three times for 5 min each, and then supplemented with 5 *μ*l of Annexin V-FITC (Beyotime) and 5 *μ*l of PI dye solution (Beyotime). The mixture was gently mixed and then incubated for 15 min at room temperature (RT, 20–30 °C). Finally, the apoptosis rates of the cells were assessed *via* flow cytometry (Agilent).

### Hochest and ROS staining

Cells were treated with TB3 as described above. After 12 h or 24 h, they were incubated with Hoechst 33,342 (Solarbio) or ROS dye (Solarbio) for 15 to 30 min at 37 °C and then washed three times with PBS. Finally, they were photographed using an inverted fluorescence microscope (Leica).

### Immunofluorescence staining

Cell-climbing slices were prepared. After drug treatment for 24 h, cells were washed three times with PBS and then fixed in 4% paraformaldehyde (PFA) (Solarbio). Then, the cells were treated with Triton X-100 (Solarbio) for 30 min, blocked in 4% bovine serum albumin (BSA) (Solarbio) for 2 h, and incubated with AR primary antibody (1:1000, CST) at 4 °C overnight. The next day, the cells were washed with phosphate buffered saline with triton x-100 (PBST), incubated with fluorescent secondary antibody (1:1000, CST) for 2 h, and washed with PBST three times, and AR nuclear localization was determined by laser confocal detection (Leica).

### Immunohistochemistry

Tumor tissues were fixed in 4% PFA (Solarbio) at RT, embedded in paraffin, and then sectioned at a thickness of 5 to 8 *μ*m. Subsequently, the sections were dewaxed using a dewaxing solution (Solarbio) and rehydrated through a graded alcohol series. Trypsin-based (BI) antigen retrieval was then performed at 37 °C for 20 min. The sections were subsequently washed in 1x PBST, blocked with 5% BSA (Solarbio) for 1 h at RT, and washed again with 1x PBST. Following this, they were incubated overnight with antibodies against AR, and PKM2 (1:200, HUABIO) at 4 °C. Afterward, the sections were washed with PBST and then incubated with secondary antibodies for 2 h at RT. Finally, the sections were washed with PBST, stained with BDA solution (Solarbio) for 5 to 10 min, and washed with PBST. Observations were made using an inverted microscope (Leica).

### Hematoxylin–eosin staining

The tumor, heart, liver, spleen, lung, and kidney from the xenotransplant-model mice were collected. They were then fixed in 4% PFA (Solarbio), paraffin-embedded, sectioned, dewaxed using a dewaxing solution (Solarbio), and rehydrated through a graded alcohol series, as described above. Cell nuclei were stained with hematoxylin (Beyotime) for 3 to 8 min, followed by rinsing the samples with tap water, a brief treatment with 1% hydrochloric acid (Beyotime), and a final rinse with tap water for blue coloration. Cytoplasm staining was achieved by immersing the sections in eosin (Beyotime) for 1 to 3 min. Dehydration was carried out through a graded alcohol series, followed by clearing in a dewaxing solution (Solarbio). Finally, the samples were sealed using neutral glue (Solarbio) and then examined using a light microscope (Leica).

### Western blotting

Cells were washed twice with precooled PBS for 5 min each and then centrifuged at 1000*g* for 5 min. Subsequently, the supernatant was removed, and the precipitated cells were incubated with radio immunoprecipitation assay lysis buffer (Beyotime) for 30 min on ice. The sample was then treated with the SDS loading buffer (Beyotime) (at a 4:1 ratio) and heated at 105 °C for 10 min to denature the proteins. The multicolor prestained protein ladder was used as a marker (WJ103, YaMei). Afterward, the proteins were resolved *via* SDS-PAGE (Solarbio) and then transferred onto a PVDF membrane (Millipore), which was subsequently washed three times in tris-HCl buffered salt solution with tween-20 (TBST), followed by 2 h of blocking with 5% BSA (Solarbio) in TBST. Afterward, the membrane was incubated with various antibodies overnight at 4 °C (antibodies against AR and GPX4 were from CST, and those against P53, Bax, Bcl-2, NQO1, HK2, ENO3, PKM2, PLK1, CDC25C, and *β*-actin were from HUABIO; all the antibodies were used at 1:1000 dilution). The following day, the antibodies were removed and the membrane was washed three times with TBST. Subsequently, it was incubated with secondary antibodies (1:30,000, CST) for 2 h at RT. Finally, the membrane was washed three times with TBST, photographed by a Chemiluminescence imager (BioRad), and subjected to analysis by ImageJ software.

### Oxidative stress and metabolism assay

Cells were prepared and lysed as described above. All the kits were purchased by the Nanjing Jiancheng Bioengineering Institute. Their metabolic rates were assessed using the lactate dehydrogenase (LDH), GSH, malondialdehyde oxidative stress kits, and glucose, lactate, pyruvic acid metabolism kit according to the instructions of the manufacturer.

### Molecular docking

Molecular docking analysis was performed by AutoDockTool software. TB3 docking was performed with protein AR (PDB:2q7i). The chemical structure of TB3 was drawn using ChemDraw (version 18.0). Protein PDB files were downloaded at http://www.rcsb.org/pdb/.

### CETSA

CETSA was used to assess TB3 binding with AR. The VCAP cells were treated with 2.5 *μ*M TB3 at 43 °C, 46 °C, 49 °C, 52 °C, and 55 °C for 3 min; the DMSO treatment group is used as a control. For gradient of concentration, the VCAP cells were treated with TB3 (0.625 *μ*M, 1.25 *μ*M, 2.5 *μ*M, 5 *μ*M, and 10 *μ*M) at 52 °C for 3 min; the 37 °C treatment group is used as a control. All cells were balanced at room temperature (22–25 °C) for 3 min, and then three cycles of rapid temperature changes, alternating between liquid nitrogen (Yagang Gas Co, Ltd) and a water bath at 37 °C. Finally, a western blot was performed to assess AR protein level which binding with TB3.

### Protein half-life experiment

Cycloheximide (CHX) inhibits the synthesis of all proteins and is used to detect the half-life of protein degradation. TB3 promoted the degradation of AR, and if the synthesis of AR protein was inhibited by CHX (50 μg/ml) (Glpbio), the simultaneous treatment of TB3+CHX accelerated the degradation of AR. Proteins treated with CHX alone and TB3+CHX together were collected at 0 h, 1 h, 6 h, 12 h, and 18 h, and AR degradation analysis was performed by western blot assay.

### Rescue assay

The cells were cultured in a 24-well plate. The G-6-P (Macklin), fructose-1, 6-diphosphate (FPD) (Macklin), phosphoenol pyruvic (Sigma), pyruvic acid (Sigma), and DHT (Glpbio) were respectively added into the cells with 2 *μ*M TB3. After 24 h, the MTT assay (Beyotime) was performed. The treatment concentration of the intermediate product was determined by MTT assay of concentration gradient experiment in a 96-well plate.

### Statistical analysis

GraphPad Prism (version 7.0) software was used to analyze all data. All data are the mean ± SD with three dependent experiments, and the one-way ANOVA was used for comparison between multiple groups. *p* < 0.05 was considered statistically significant.

## Ethics statement

No human participants were involved in this study, and all the animal experiments were approved by the Institutional Animal Care Committee of the Guizhou Medical University (approval no: 2304063, validity period: March 22, 2023–March 22, 2024).

## Data availability

Data will be made available on request.

## Supporting information

This article contains supporting information.

## Conflict of interest

The authors declare that they have no conflicts of interest with the contents of this article.

## Supporting information

Supporting Figure S1 **TB3 directly binds to AR in CRPC cells.***A*, venn diagram of the target genes predicted using SwissTargetPrediction and the Genecards, Disgenet, and OMIMG databases. *B*, protein–protein interaction networks analysis of the 28 key targets, as assessed using the String website. *C*, delta-G energies of the top 10 candidate target genes, as assessed using AutodockTools. *D*, molecular-docking results based on AutodockTools.
