# Supplementary figures and images for: Tanshinone analog inhibits castration-resistant prostate cancer cell growth by inhibiting glycolysis in an AR-dependent manner
Source: J Biol Chem. 2024 Mar 5;300(4):107139. doi: 10.1016/j.jbc.2024.107139 (PMC11002303; doi:10.1016/j.jbc.2024.107139)

# Figure S1

A

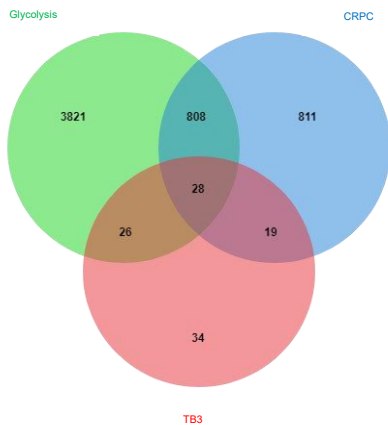

B

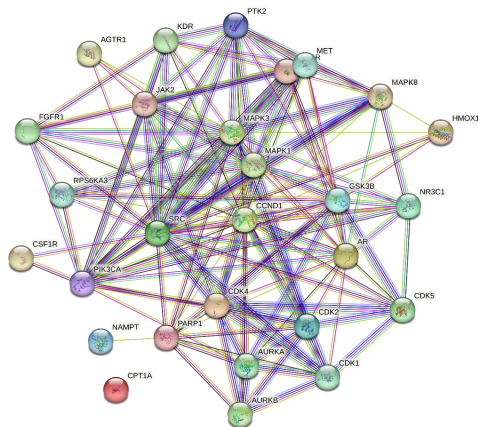

C

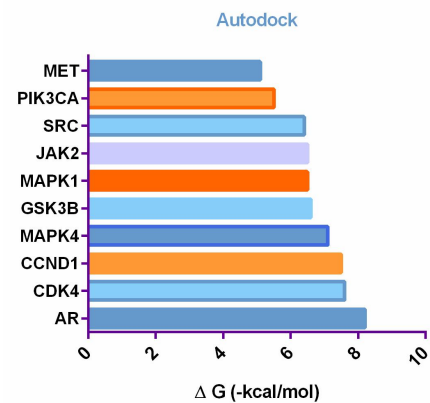

D

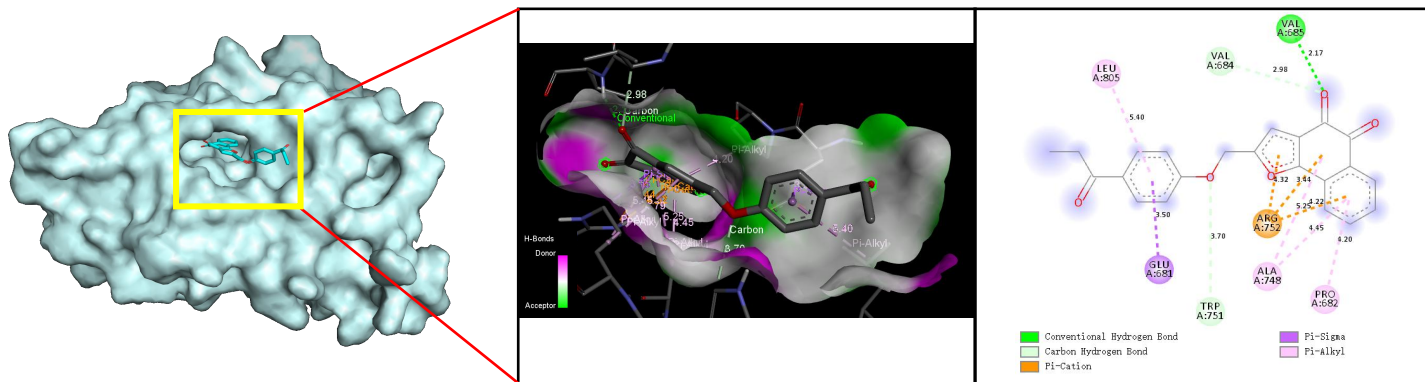

Supplement: Supporting Figure S1 — TB3 directly binds to AR in CRPC cells.A, venn diagram of the target genes predicted using SwissTargetPrediction and the Genecards, Disgenet, and OMIMG databases. B, protein–protein interaction networks analysis of the 28 key targets, as assessed using the String website. C, delta-G energies of the top 10 candidate target genes, as assessed using AutodockTools. D, molecular-docking results based on AutodockTools. [file mmc1.pdf]
